# Sleep-Dependent Consolidation of Value-Based Learning

**DOI:** 10.1371/journal.pone.0075326

**Published:** 2013-10-09

**Authors:** Bengi Baran, Dasha Daniels, Rebecca M. C. Spencer

**Affiliations:** 1 Department of Psychology, University of Massachusetts, Amherst, Amherst, Massachusetts, United States of America; 2 Neuroscience and Behavior Program, University of Massachusetts, Amherst, Amherst, Massachusetts, United States of America; Central Queensland University, Australia

## Abstract

It has been suggested that sleep selectively enhances memories with future relevance. Given that sleep’s benefits can vary by item within a learning context, the present study investigated whether the amount of sleep-dependent consolidation may vary across items based on the value of the to-be-learned material. For this purpose, we used a value-based learning paradigm in which participants studied words paired with point values. There were two groups; participants either studied the words in the evening and were tested after a 12 hr interval containing a full night of sleep, or studied the words in the morning and were tested after 12 hr of continuous daytime wake. Free recall (F(1,36) = 19.35, *p*<.001) and recognition accuracy (F(1,36) = 7.59, *p* = .01) for words were better following sleep relative to wake. However there was no difference in the linear increase in the probability of delayed recall with increasing word value for sleep and wake groups (*p* = .74). Thus, while encoding may vary with the value of the to-be-learned item, sleep-dependent consolidation does not.

## Introduction

Sleep enhances declarative memory consolidation [1]. Performance on several tasks is better if learning is followed by an interval with sleep compared to an equal amount of time spent awake. Moreover, changes in memory performance over sleep correlate with specific physiological components of sleep. Over-sleep consolidation of declarative learning is associated with time spent in slow wave sleep (SWS), a deep stage of sleep marked by highly synchronous delta activity [2], [3] and hippocampal activation [4]. Animal studies revealed that memory consolidation during sleep occurs via neural reactivation; firing patterns associated with waking experiences are replayed during sleep [5], [6].

Memory benefits can be accounted for with the Synaptic Homeostasis Hypothesis. Accordingly, the function of sleep is to regulate the increased synaptic potentiation that occurs during the day [7]. Specifically, synaptic strength in cortical circuits increases throughout the day due to learning. Over sleep, this heightened synaptic strength is downscaled. Homeostatic regulation occurs via global synaptic downscaling that results in pruning of weaker synaptic connections.

A series of recent studies suggest that memories can be selectively consolidated or forgotten over sleep. In one such study, retrieval of word pairs was tested following intervals that contained overnight sleep, nighttime wake (i.e. sleep deprivation), and daytime wake [8]. Sleep-dependent performance enhancement was observed only if the participants were informed that recall would be tested after the 9 hr delay interval. Recall following sleep and wake did not differ for groups who performed a surprise delayed recall test. In other words, a sleep benefit was observed only if the learned material carried an expected future relevancy (i.e. an upcoming test).

One way of probing future relevance is associating learning with a future reward. Fisher and Born [9] trained participants on two different versions of a motor sequence learning task. Participants were instructed that performance for only one of the sequences was associated with a monetary reward. Performance for both sequences was better following a 12 hr interval containing overnight sleep compared to performance following daytime wake. Importantly, however, performance changes following sleep were significantly greater for the rewarded than the non-rewarded sequence.

These findings suggest that memory consolidation over sleep may be prioritized for information that carries future relevance. However, in both studies future relevance was either absent or present. The focus of the present investigation is to determine whether sleep-dependent memory consolidation changes as a function of increasing future relevance of the to-be-learned material. Recently, Oudiette and colleagues [10] used an object-location association task in which objects were assigned a high or low value. Recall of object locations was tested in all groups after an interval that either included a 90-min afternoon nap or an equal interval in which participants remained awake. The authors found that recall accuracy declined significantly more for low-value objects than high-value objects. Contrary to previous studies with object-location association tasks [11]–[14], accuracy was similar following sleep and wake suggesting that a nap did not benefit learning. However, using task-related cues (i.e. sounds characteristic to the studied objects) to reactivate a subset of object-location associations during SWS, recall of low-value associations was enhanced relative to recall of objects not reactivated and similar to recall of high-value associations.

In the present study we examined whether there are increases in consolidation with linear increases in item value using a value-based learning task e.g. [15]. Given that all items in this paradigm have future relevance (value), one might posit that all are equally consolidated over sleep [8]. If such is the case, one would expect no difference in the slope of the value-recall relationship following sleep and wake. Alternatively, given that the point value varies across items, those with the highest value may be prioritized for consolidation over those with less reward value. Previous studies suggest that sleep can act differently on individual items learned within the same context [13]. Thus, the slope of the value-recall relationship could differ for the sleep and wake conditions, with sleep-dependent consolidation increasing linearly as a function of increasing item value. Here we examined these alternatives by examining recall of a value-based learning task following intervals with sleep and wake.

## Materials and Methods

### Ethics Statement

Testing procedures were approved by the University of Massachusetts, Amherst Institutional Review Board and written informed consent was obtained prior to the experiment. Participants received course credit for participation.

### Participants

Participants were 38 young adults (25 female, 13 male), 18–30 years of age (mean = 20.29 years, SD = 2.04). All participants were native English speakers with normal or corrected-to-normal vision. Exclusion criteria included history or presence of a neurological, psychiatric or sleep disorder; habitual overnight sleep of <6 hr; and use of medication known to affect sleep or cognition. Prospective participants completed an in-house screening questionnaire to determine eligibility.

### Task and Procedures

Participants were assigned to one of two groups and were tested over two sessions separated by a 12 hr interval. The Sleep group (n = 18) completed the first session in the evening (starting between 7–10 p.m.) and the second session the next morning (starting between 7–10 a.m.). The Wake group (n = 20) completed their first session in the morning (starting between 7–10 a.m.) and the second session in the evening (starting between 7–10 p.m.). Participants were asked to refrain from consuming alcohol or more than one 12 oz of coffee or caffeinated beverage for the duration of the experiment. Participants in the Wake group were asked to refrain from napping. Otherwise, participants were allowed to maintain their daily routine.

At the beginning session 1, participants completed the first part of an in-house sleep and daily activities diary. Subsequently, participants began the value-based learning task. This task is similar to that described elsewhere [15]. A total of 120 four-letter English nouns were divided into 6 lists. Lists were matched for word-frequency [16]. The 20 words on each list were randomly paired with a point value ranging from 1–20.

The first session consisted of 6 learning blocks. In each block, participants viewed 20 word-point value pairs. Each pair was presented for 1000 ms with an inter-stimulus interval of 500 ms. Following Castel and colleagues [15] participants were instructed: “Your task is to try to get as many points as possible and this can be accomplished by remembering as many of the high value words as you can”. After viewing all 20 pairs, participants performed a distracter task (backward serial counting) for 3 min to avoid recency effects on immediate recall. Following the distracter task, immediate recall was probed by asking participants to verbally report all the words they could remember. At the end of immediate recall participants were told their score (i.e. the sum of point values of the words they recalled) and were once again encouraged to get the highest score possible. This block of encode-distractor-immediate recall cycle was repeated for each of the six word-value lists. At the end of the first session, participants completed the Pittsburg Sleep Quality Index (PSQI) and the Epworth Sleepiness Scale (ESS). The PSQI is a measure of habitual sleep quality over the last 30 days [17]. The ESS provides a measure of general sleepiness [18].

The second session started with completing the in-house sleep and daily activities diary. Subsequently, participants performed three memory probes: free recall, cued recall and recognition. In the delayed free recall probe, participants were instructed to recall as many words as they could from the first session irrespective of list. No feedback was provided. Free recall was followed by a recognition test. Sixty words from the first session were intermingled with 60 new words (matched in word frequency to target items). Participants were asked to make a Yes/No judgment about whether they had seen the word in the previous session. Finally, participants completed a cued-recall probe. Sixty words from the first session (that were not shown in the recognition task) were presented individually and participants were asked to recall the point value it was paired with. Participants could respond by providing a specific number, a range (comprised of values between 1–5, 6–10, 11–15 or 16–20), or they could choose a “don’t remember” option. All memory probes were self-paced.

### Statistical Analyses

Free recall for the first session was measured as total number of words correctly recalled over 6 learning blocks. Accordingly, recall value was measured as the sum of point values for correctly recalled words. Free recall and recall value scores for the second session were calculated in the same manner. Next, for each participant, free recall performance was adjusted to immediate recall performance by computing an adjusted recall score:
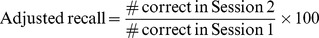



Of particular interest is whether recall linearly increases with the value of the word and whether this slope differs for Sleep and Wake groups. We used a mixed-effects logistic regression [19] as implemented in the lme4 package [20] of the R statistical programming language [21] to investigate the relationship between point value of the words (1–20) and group (Sleep vs. Wake) with probability of recall in Session 1 or Session 2 as the dependent variables.

Recognition memory was measured by calculating the discriminability measure, d’. Two measures were calculated for value recall based on how the participants chose to report: accuracy for specific reported responses and accuracy for range responses. Unless otherwise noted all group comparisons were made by univariate ANOVAs with group (i.e. Sleep or Wake) as the between subjects factor. For all of the analyses, effect sizes are reported as η_p_^2^.

## Results

### Sleep Characteristics

Sleep and Wake group participants were similar on measures of daytime sleepiness (ESS total score), habitual sleep quality (total PSQI score), subjectively estimated habitual total sleep duration and habitual sleep latency over the last 30 days (see Table 1). The Sleep group participants slept an average of 6.66 hrs (SD = 1.31) on the experimental night as reported in sleep diaries. Subjectively reported sleep time did not predict immediate (r = −.18, p = .49) or delayed recall (r = .20, p = .44) performance.

**Table 1:** Participant Characteristics.

	Sleep (n = 18)		Wake (n = 20)		
	mean	SD	mean	SD	*p* *
Age (yrs)	19.89	1.18	20.65	2.56	.26
ESS	7.78	4.15	7.80	3.68	.99
PSQI	4.83	2.57	5.85	2.37	.21
Habitual sleep time(hrs)	7.65	.85	7.52	1.14	.71
Habitual sleep latency (mins)	17.89	9.47	20.55	12.44	.47

* *p* values are for one-way ANOVA tests, F(1,36); ESS: Epworth Sleepiness Scale; PSQI: Pittsburg Sleep Quality Index.

### Immediate Recall

**Figure 1:**
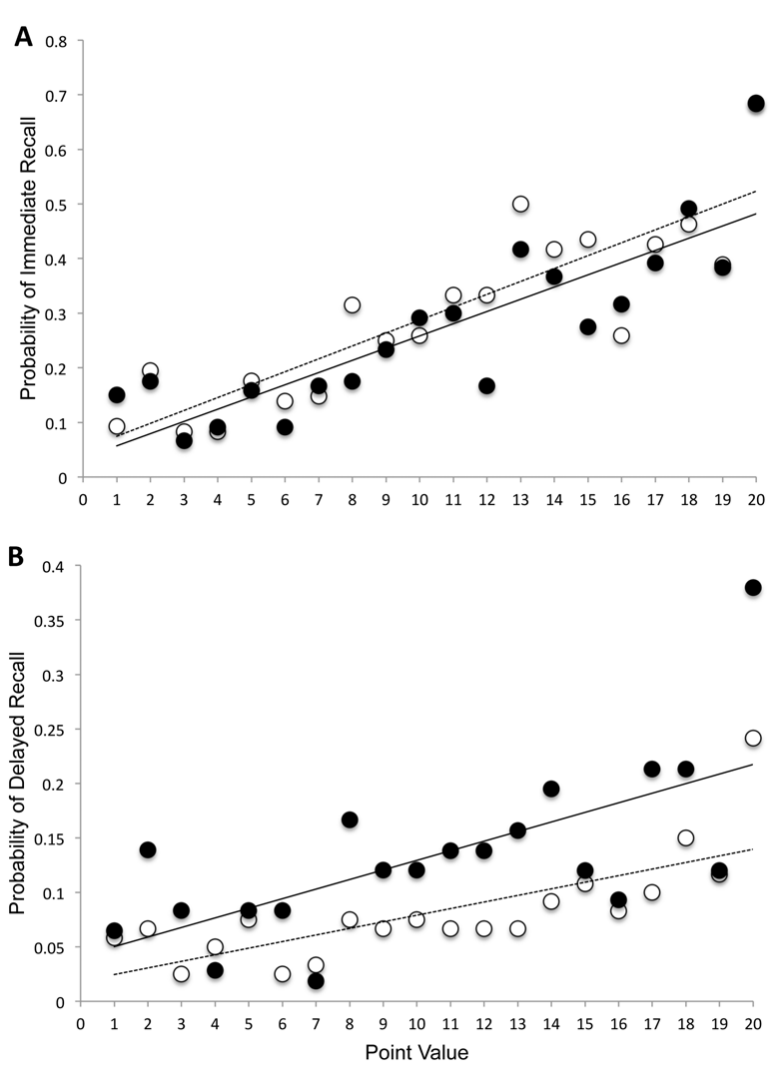
Recall as a function of point value. Probability of immediate (A) and delayed (B) recall as a function of point value of the word separated for Sleep and Wake groups (black circles = sleep group; white circles = wake group). Lines represent linear regression (solid = Sleep; dashed = Wake). Note change of scale.

Figure 1A illustrates the probability of immediate recall by item value. Consistent with Castel and colleagues [15], immediate recall increased with increasing value of the item to be learned. In order to investigate possible baseline differences or time-of-day effects on encoding, immediate recall was compared across groups. An ANOVA on the number of words correctly recalled with group (Sleep vs. Wake) as the between subjects factor revealed no significant group effect, F(1,36) = 1.66, *p* = .21, η_p_^2^ = .04. The same analysis was run for recall value (i.e. sum of point values for words successfully recalled). Here, too, we did not observe a significant difference between the Sleep and Wake groups, F(1,36) = 1.13, *p* = .29, η_p_^2^ = .03. These results suggest that irrespective of whether learning occurred in the morning or the evening, initial learning performance was similar across groups.

### Delayed Free Recall

**Figure 2:**
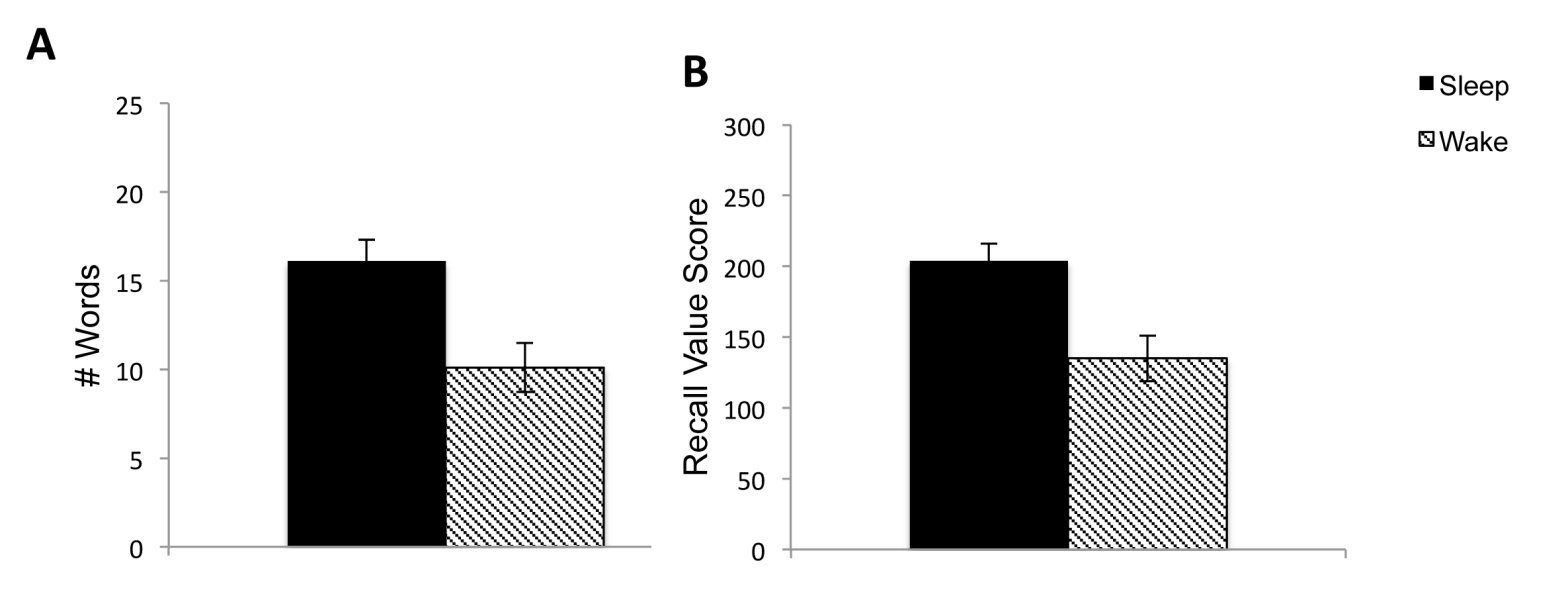
Delayed Free Recall. Results for the delayed free recall task. A) Total number of words recalled on Session, B) Total recall score (sum of point values of words correctly recalled). Error bars indicate standard error of the mean.

Total number of words recalled in the delayed free recall task was compared between participants in the Sleep and Wake groups. Recall performance was better following sleep compared to wake, F(1,36) = 11.29, *p* = .002, η_p_^2^ = .24 (Fig. 2A). An ANOVA on adjusted recall (i.e. delayed recall adjusted for immediate recall performance) revealed a significant effect of group, F(1,36) = 19.35, *p*<.001, η_p_^2^ = .35, with the Sleep group performing better than the Wake group.

Recall value, defined as the sum of point values for words correctly recalled in delayed free recall, was also significantly higher for the Sleep group than the Wake group, F(1,36) = 10.82, *p* = .002, η_p_^2^ = .23 (Fig. 2B). To examine whether recall value was greater for the Sleep group because more items were recalled, we calculated the average point value for recall in each group. With a repeated measures ANOVA we found no main effect of group (Sleep vs. Wake), F(1,36) = .01, *p* = .92, η_p_^2^<.001, no main effect of session (Immediate vs. Delayed), F(1,36) = 1.31, *p* = .32, η_p_^2^ = .03 and no significant interaction, F(1,36) = .004, *p* = .95 η_p_^2^<.001. Thus, greater recall in the Sleep group resulted in higher total points associated with recall rather than greater recall of higher value items per se.

Figure 2B shows probability of recall as a function of point value of the words. In order to determine whether the relationship between probability of recall and value of the word is different for Sleep and Wake groups we used a mixed-effects logistic regression. There was a main effect of point value, with the log odds of correctly recalling a word increasing by 0.08 for each increment in point value, SE = .01, z = 6.06, *p*<.001. There was also a main effect of group with higher probability of correct recall in the Sleep group, SE = .17, z = 3.51, *p*<.001. However, there was no significant interaction, *p* = .74 suggesting that the relationship between point value and probability of recall was similar when recall was completed after an interval of overnight sleep or after continuous daytime wake.

### Recognition Accuracy (*d’*)

**Figure 3:**
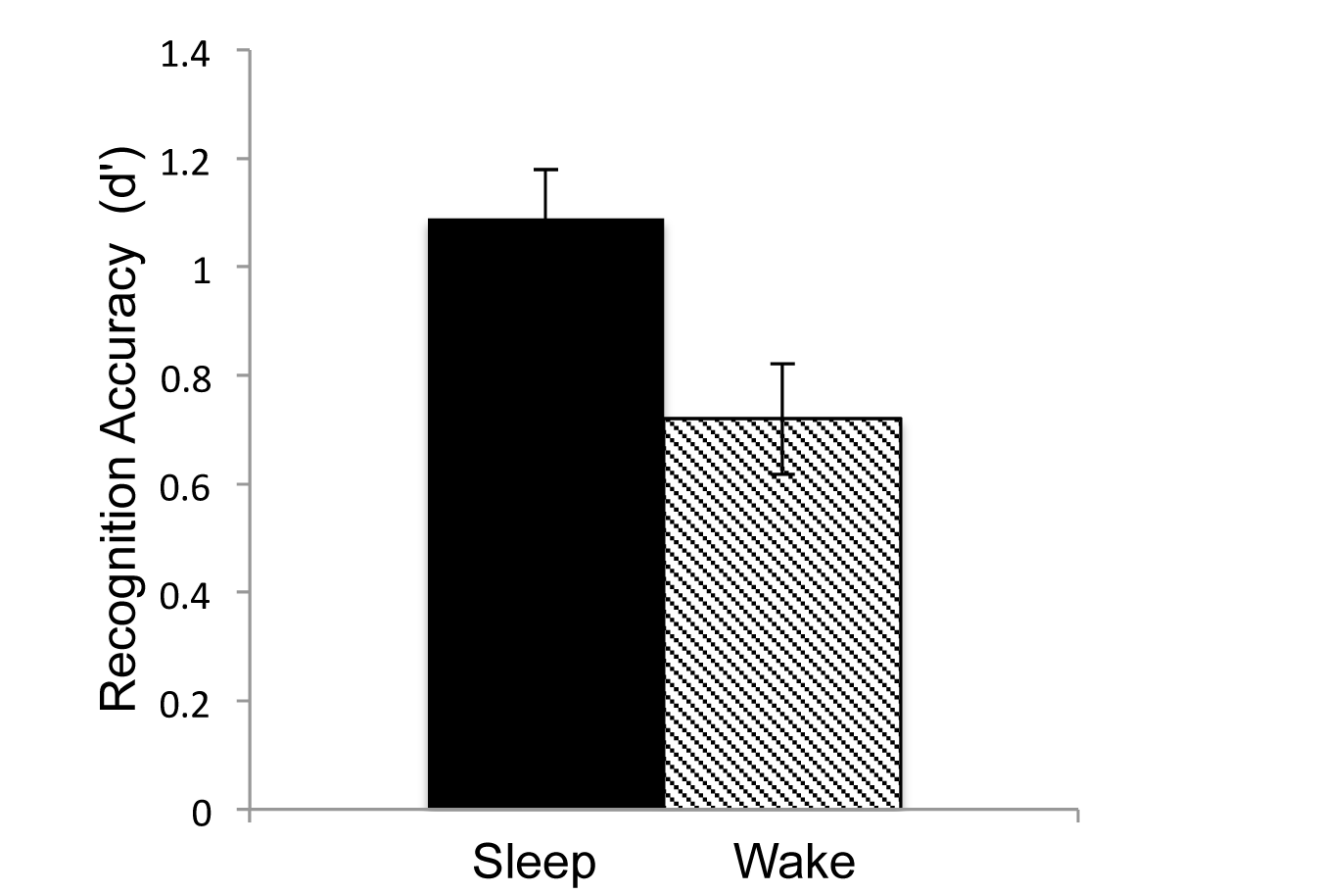
Recognition. Recognition accuracy as measured with d’. Error bars indicate standard error of the mean.

An ANOVA on d’ showed that recognition accuracy was better in the Sleep group than the Wake group, F(1,36) = 7.59, *p* = .01, η_p_^2^ = .19 (Fig. 3).

Of interest, again, was whether sleep enhances recognition accuracy as a function of point value. Importantly, since only the target words have been assigned a point value, this analysis is limited to Hit Rates (i.e. correctly recognized target words). Logistic regression analysis revealed a significant main effect of point value with the log odds of correctly recalling a word increasing by 0.097 for each increment in point value, SE = .01, z = 7.45, *p*<.001. There was no significant main effect of group suggesting that hit rate was similar between Sleep and Wake groups, *p* = .28 and no significant interaction between point value and group, *p* = .43.

### Value Recall

Two measures were calculated for value recall based on how the participants chose to report: accuracy for specific reported responses and accuracy for range responses. Participants reported 69.6% of the words as a range value and Sleep and Wake group participants were similarly likely to assign range responses, F(1,36) = 0.62, p = .44, η_p_^2^ = .02. Five percent of the words were assigned a specific value and Sleep and Wake group participants were similarly likely to assign specific responses, F(1,36) = 0.96, p = .33, η_p_^2^ = .03. Of the items participants provided specific responses for (e.g., given a word, chose to state an exact point value), accuracy was significantly higher in the Sleep group, Sleep mean = 52%, SEM = 15.1%; Wake mean = 19%, SEM = 6.5%; F(1, 25) = 4.89, *p* = .04, η_p_^2^ = .16. Accuracy for range responses (1–5, 6–10, 11–15 or 16–20) revealed a non-significant trend for better performance in the Sleep group, Sleep mean = 33%, SEM = 1.5%; Wake mean = 29%, SEM = 1.6%; F(1, 36) = 3.250, *p* = .09, η_p_^2^ = .08.

## Discussion

In accordance with previous research [2], [3], we provide evidence that sleep enhances declarative memory retrieval. Participants who slept in the intersession interval had better recall and recognition accuracy than those who stayed awake. Future relevance of the to-be-learned material was manipulated by assigning a numerical point value to each word and instructions to obtain the highest points possible. Therefore, we investigated whether sleep-dependent memory consolidation increases as a function of increasing future relevance. A logistic regression of recall probability by value revealed that sleep had a similar benefit on recall across the range of values. This is clear in Figure 1. Thus, sleep may prioritize all items with future relevance for consolidation equally.

Importantly, there was no circadian influence on performance. First, there was no difference between groups in initial learning performance. Both groups performed similarly in immediate recall in terms of the number of words recalled and total recall value suggesting that initial encoding strategies were similar regardless of whether encoding took place in the morning (Wake group) or evening (Sleep group). Second, similar results were found when session 2 recall was adjusted to account for individual differences in immediate recall.

In this value-based learning paradigm, all items were associated with a point value. Meaning, all items had a ‘future relevance’. Consistent with Wilhelm and colleagues [8], sleep enhanced the probability of recalling these items with future relevance. Without items or a condition in which no value was assigned, our results cannot speak to whether future relevance/value is a necessary condition for sleep-dependent consolidation. Rather, we emphasize here that consolidation of memories with future relevance is not biased by the amount of value. It is possible, however, that all items were consolidated equally over sleep by being learned in the same context. While this cannot be ruled out, previous studies suggest that items learned in the same context can be differentially consolidated. For instance, Rudoy and colleagues [13] had participants encode the location of items in conjunction with a related cue (‘meow’ with an image of a cat), and recall after sleep was greater for those items for which the sound cues were replayed during sleep.

Consistent with prior work on value-based learning in young adults, participants in both the Sleep and Wake conditions were more likely to recall words with high values than words with low values. Healthy older adults compensate for reduced overall memory by preferentially encoding, via selective attention, high-value items [15]. Older adults with Alzheimer’s disease [22] and children with attention-deficit hyperactivity disorder [23] show reduced value-based selectivity. Importantly, in this corpus of work, greater recall of high-value words is assumed to reflect strategic learning in the encoding stage as opposed to the recall stage of memory. If such is the case, then the present results suggest that the scale of sleep-dependent consolidation is independent of the amount or strength of initial learning. Consistent with this, we have shown that initial learning does not correlate with oversleep consolidation (intersession change in recall) in young or older adults [24]. Wilhelm and colleagues [25] manipulated the amount of initial learning by providing young adults either 2 blocks or 10 blocks of training on the motor sequence learning task. Contrary to the present results, in this study the over-sleep reduction in reaction time (the measure of learning on this task) was greatest for those with low initial learning (2 blocks of training) compared to those with high initial performance (10 blocks of training). This may reflect that high performers approached ceiling performance and had limited room for improvement relative to low performers. Alternatively, the relationship between encoding and consolidation may differ for procedural and declarative learning tasks.

In the present study salience manipulation was based on instructions to the participant to try to receive high scores. This may be a potential limitation as there was no objective incentive. Adjusting participants’ compensation based on task performance may increase salience and such a design may be of interest for future studies. Notably, however, Wilhelm and colleagues [8] manipulated consolidation simply by stating future relevance without a corresponding reward. Nonetheless it is possible that a financial reward could enhance this manipulation.

Furthermore, as we aimed to compare retention of value-based learning over nocturnal sleep and daytime wake in the most naturalistic setting, we did not have control of participants’ daytime activities that may potentially interfere with consolidation. For the same reason, sleep was only subjectively measured. Subsequent studies would gain from polysomnography-recorded sleep so the unique or opportunistic role of sleep can be more thoroughly evaluated.

In conclusion, we demonstrate sleep-dependent consolidation of a declarative learning task that is not preferential or selective for items with the greatest value. This result may seem to contradict the theory posed by us [1] and others [26], which posits that memories are filtered over sleep, selectively enhancing memories that have emotional salience or future relevance. Rather, we consider these results in support the role of future relevance as a broad memory filter but define a limitation on how restrictive this filter is.
